# The mediating effect of dysmorphic concern in the association between avoidant restrictive food intake disorder and suicidal ideation in adults

**DOI:** 10.1186/s12888-023-05490-5

**Published:** 2024-01-10

**Authors:** Gaelle Salameh, Nour El Khoury, Rabih Hallit, Diana Malaeb, Fouad Sakr, Mariam Dabbous, Feten Fekih-Romdhane, Sahar Obeid, Souheil Hallit

**Affiliations:** 1 School of Medicine and Medical Sciences, Holy Spirit University of Kaslik, P.O. Box 446, Jounieh, Lebanon; 2 Department of Infectious Disease, Bellevue Medical Center, Mansourieh, Lebanon; 3 Department of Infectious Disease, Notre Dame des Secours University Hospital, Byblos, Postal Code 3 Lebanon; 4 College of Pharmacy, Gulf Medical University, Ajman, United Arab Emirates; 5 School of Pharmacy, Lebanese International University, Beirut, Lebanon; 6 The Tunisian Center of Early Intervention in Psychosis, Department of Psychiatry “Ibn Omrane”, Razi Hospital, 2010 Manouba, Tunisia; 7 Faculty of Medicine of Tunis, Tunis El Manar University, Tunis, Tunisia; 8 School of Arts and Sciences, Social and Education Sciences Department, Lebanese American University, Jbeil, Lebanon; 9 Applied Science Research Center, Applied Science Private University, Amman, Jordan

**Keywords:** Avoidant, Restrictive Food Intake Disorder (ARFID), Dysmorphic concerns, Suicidal ideation

## Abstract

**Background:**

Reflecting on the existing literature on suicidal ideation and Avoidant/Restrictive Food Intake Disorder (ARFID), this article investigates the complex relationship between them, hypothesizing about the possibility of dysmorphic concerns, being a mediator linking ARFID to suicidal ideation.

**Methods:**

Using a snowball sampling approach, a survey was created on Google Forms and circulated across messaging applications and social media networks (WhatsApp, Instagram, Messenger). The sample involved 515 participants recruited between February and March 2023. The questionnaire included the following scales: Nine-items Avoidant/Restrictive Food Intake Disorder screen (NIAS), Dysmorphic Concern Questionnaire (DCQ), and Columbia-Suicide Severity Rating Scale (C-SSRS). When filling the questionnaire, respondents were warned that they can experience distress when answering certain questions and received information about mental health services. Five hundred fifteen adults participated in this study, with a mean age of 27.55 ± 10.92 years and 60.1% females.

**Results:**

After adjusting over potential confounders (i.e., age, education, marital status, and household crowding index), analyses showed that dysmorphic concerns fully mediated the association between avoidant restrictive eating and suicidal ideation. Higher avoidant restrictive eating was significantly associated with more dysmorphic concerns, and higher dysmorphic concerns were significantly associated with the presence of suicidal ideation. Finally, avoidant restrictive eating was not significantly associated with suicidal ideation.

**Conclusion:**

This study highlights the potential indirect link between ARFID and suicidal ideation mediated by dysmorphic concerns. While no direct connection was observed between ARFID and suicidal ideation, the presence of dysmorphic concerns appeared to be a crucial factor in amplifying the risk of suicidal ideation in individuals with ARFID. This emphasizes the importance of addressing dysmorphic concerns alongside ARFID treatment to enhance mental health interventions and outcomes.

## Introduction

Suicide is defined as a fatal self-harming act that is meant to end one's life. In 2015, the age-standardized suicide rate worldwide was 12 per 100,000 individuals [1]. It was considered the 14th leading cause of overall death [1]. Suicidal ideation is described as having ideas about committing suicide, some of which may involve a plan. The World Health Organization (WHO) carried out community surveys in 21 countries and discovered that the prevalence of suicidal ideation was 2% over a 12-month time frame [2] and 9% throughout a lifetime [3]. Among those with a history of suicidal thoughts, the likelihood that they will ever make a plan is around 33%, and the likelihood that they will ever commit suicide is around 30% [3].

Suicidal ideation and behavior are complex multifactorial phenomena with a wide range of genetic, biological, psychological, psychiatric, economic, cultural, and social factors being involved [3], including the presence of mental disorders. As such, it is crucial to consider conditions like eating disorders, as they can greatly influence emotional well-being and potentially intersect with discussion surrounding suicide prevention. Among eating disorders is the avoidant/restrictive food intake disorder (ARFID) [4].

### The association between ARFID and suicidal ideation

According to the DSM-5-TR [5], ARFID is defined according to the following criteria: first, avoidance or restriction of dietary consumption, which may be due to lack of desire for food, an avoidance of food because of its unpleasant sensations, or a fear of unpleasant consequences of eating like vomiting and choking; second, consistent inability to meet nutritional and/or energy needs due to limitation on the quantity or types of foods consumed; third, an absence of food shortage or culturally sanctioned practice as the cause of the eating problem; fourth, an absence of body weight and shape distortion with the disruption not being limited to anorexia nervosa or bulimia nervosa; and fifth, neither a medical disease nor another psychiatric illness can explain the symptoms [5]. ARFID can develop at any weight, however, people suffering from ARFID are frequently underweight [6]. In the general population, ARFID’s prevalence was found to range from 0.3% in people aged 15 and older [7], to 3% in younger people aged 8 to 13 years [8].

Research has shown a strong link between eating disorders and suicidal ideation [9]. Although we could find no previous study directly linking ARFID to suicidal ideation, one paper showed that individuals with acute ARFID symptoms (< 12 months) reported a higher number of suicidal outcomes compared to those with chronic ARFID symptoms (≥ 12 months) [10]. In light of the existing literature on ARFID and suicidal ideation, it is plausible to consider a possible indirect factor that could associate ARFID and suicidal ideation. In this paper, it is proposed to add to the existing literature by examining, for the first time, this relationship and possible mediators implicated in it. Among possible mediating factors, dysmorphic concerns may play a crucial role.

### Body dysmorphic concerns as mediator

Body dysmorphic concerns (BDC) refer to concerns with nonexistent or minor physical flaws, leading patients to feel they are abnormal, ugly, or deformed while in fact, they are normal looking. The obsession with perceived imperfections causes repetitive actions, such as checking one's reflection in a mirror, that are disagreeable and hard to control [5]. BDC are distinguishable from the distorted perceptions of body shape and weight [11]. Similarly, ARFID is not related specifically with body weight or shape disturbance experiences. There is evidence that ARFID may co-exist with BDC (e.g., [12]). Indeed, psychiatric comorbidities are known to be prevalent in ARFID patients. In an analysis of 74 young people with ARFID, 45% had a minimum of one psychiatric comorbidity [13].

On the other hand, an investigation showed that depression, one of the main causes of suicide [14] can be caused by BDC and self-hate, which may, in turn, lead to loss of appetite and profound malnutrition eventually resulting in the diagnosis of ARFID [12]. Furthermore, body dysmorphic symptoms have consistently been shown to be linked to a potential risk of suicidal ideation in adolescence and emerging adulthood [15]. Alarmingly high rates of suicidality were also reported in patients diagnosed with body dysmorphic disorders according to meta-analysis findings, with an increase in suicidal ideation risk estimated at approximately four-fold [16]. These associations in the literature can lead us to discuss the importance of studying the mediating role of BDC between ARFID and suicidal ideation.

### The present study

Given that suicidal ideation is a significant public health concern globally and because there is a possible indirect link between ARFID and suicidal ideation, which studies did not elucidate yet, we decided to conduct a study aiming to explore the effect of dysmorphic concerns as a mediator in the association between ARFID and suicidal ideation. Our study will help fill a gap in the literature and contribute to the improvement of mental health outcomes by promoting a better understanding of the mechanisms linking ARFID and suicidal ideation. Furthermore, by investigating the role of dysmorphic concerns as a potential mediator, we seek to provide clinicians and mental health practitioners with valuable insights for designing targeted interventions that address both ARFID-related distress and the risk of suicidal thoughts in individuals affected by this disorder. The hypothesis for this study proposes that dysmorphic concerns may serve as an indirect factor linking ARFID to a higher risk of suicidal ideation.

## Methods

### Study design

Using a snowball sampling approach, a survey was created on Google Forms and circulated across messaging applications and social media networks (WhatsApp, Instagram, Messenger). The sample involved 515 participants recruited between February and March 2023. Inclusion criteria for participation included: (1) being of a resident and citizen of Lebanon, (2) aged 18 years and above, (3) having access to the Internet, and (4) willing to participate in the study. Excluded were those who refused to fill out the questionnaire. Internet protocol (IP) addresses were examined to ensure that no participant took the survey more than once. After providing digital informed consent, participants were asked to complete the instruments described above, which were presented in a pre-randomized order to control for order effects. The survey took approximately 20 min on average to be completed [17]. Ethics approval for this study was obtained from the ethics committee of the School of Pharmacy at the Lebanese International University (2023RC-014-LIUSOP). Participants were informed about the aims and nature of our work and provided written consent for the collection of their data for research purposes. The study was conducted on a voluntary basis, without remuneration. Anonymity and confidentiality were respected. Respondents were warned about possible distress that can be caused by the questionnaire and received information about mental health services.

### Minimal sample size calculation

A minimal sample of 411 was deemed necessary using the formula suggested by Fritz and MacKinnon [18] to estimate the sample size: $n=\frac{L}{f2}+k+1$, where f = 0.14 for small effect size, L = 7.85 for an α error of 5% and power β = 80%, and k = 9 variables to be entered in the model.

### Questionnaire

The questionnaire comprised questions about sociodemographic details such as age, sex, education level, living area, marital status and Household Crowding Index (HCI), which is calculated by dividing the total count of people living in a household by the total number of rooms in that household, excluding the kitchen [19]. Participants were questioned about their height and weight to obtain their Body Mass Index (BMI) [20]. The following scales were used:

*Nine-items Avoidant/Restrictive Food Intake Disorder screen (NIAS)* [21] is a validated scale in Arabic [17] that was designed to screen for ARFID. It is composed of 9 items, scored on a 6-point Likert scale, “Strongly disagree,” “Disagree,” “Slightly disagree,” “Slightly agree,” “Agree,” and “Strongly agree”. The NIAS is comprised of three subscales: the picky eating subscale measures sensory aversion to food (e.g., *“I dislike most foods that other people eat”*), the appetite subscale measures a lack of interest in eating or food (e.g., “*Even when I am eating foods I really like, it is hard for me to eat a large enough volume at meals”*), and the fear subscale measures fear of aversive consequences as a consequence of eating (e.g., *“I avoid or put off eating because I am afraid of GI discomfort, choking, or vomiting”*). Subscales are each scored on a scale from 0–15, with higher scores indicating higher levels of each metric (picky eating, lack of interest, and fear). All items may also be summed to calculate a total score, ranging from 0–45, with higher scores indicating higher levels of avoidant/restrictive eating broadly [21] (Cronbach’s alpha in this study = 0.88).

*The Dysmorphic Concern Questionnaire (DCQ)* comprises seven self-report items and is a validated tool [22] designed to assess an individual's degree of dysmorphic concern. Respondents rate their level of distress and concern on a Likert-type scale, ranging from "Not at all" to "Extremely." Scores are calculated by summing the item ratings, with higher scores indicating greater body dysmorphic concern. Example items inquire about distress regarding facial appearance, interference with daily life, and frequency of appearance-related thoughts (e.g. Have you ever been very concerned about some aspect of your physical appearance) [23]. (Cronbach’s alpha in this study = 0.89).

*The Columbia-Suicide Severity Rating Scale (C-SSRS)* is a structured assessment tool used to evaluate the severity of suicidal ideation and behavior in individuals [24]. It is validated in Arabic for both adolescents [25] and adults [26]. It employs a set of questions to gauge the intensity and frequency of suicidal thoughts and actions. The scale provides a framework for categorizing the severity of suicidal ideation and behavior, ranging from "Wish to be dead" to "Actual attempt." Example items include probing for the presence of passive thoughts about death, active suicidal ideation, and past suicide-related behaviors. (Cronbach’s alpha in this study = 0.79).

### Statistical analysis

The SPSS software v.25 was used for the statistical analysis. The suicidal ideation score and its LOG transformation were not normally distributed; therefore, the score was dichotomized into absence and presence of suicidal ideation. The Chi-square test was used to compare two categorical variables and the Student t test to compare two means. The mediation analysis was conducted using PROCESS MACRO (an SPSS add-on) v.3.4 model 4; four pathways derived from this analysis: pathway A from the independent variable to the mediator, pathway B from the mediator to the dependent variable, Pathway C’ indicating the direct effect from the independent to the dependent variable. Results were adjusted over all variables that showed a *p* < 0.25 in the bivariate analysis. We considered the mediation analysis to be significant if the Boot Confidence Interval did not pass by zero. *P* < 0.05 was deemed statistically significant.

## Results

### Sociodemographic and other characteristics of the sample

Five hundred fifteen adults participated in this study, with a mean age of 27.55 ± 10.92 years and 60.1% females. Other descriptive statistics of the sample can be found in Table 1.

**Table 1:** Sociodemographic and other characteristics of the sample (*N* = 515)

Variable	N (%)
Sex	
Male	155 (30.1%)
Female	360 (69.9%)
Marital status	
Single	377 (73.2%)
Married	138 (26.8%)
Education	
Secondary or less	84 (16.3%)
University	431 (83.7%)
Living area	
Urban	298 (57.9%)
Rural	217 (42.1%)
Suicidal ideation	
No	374 (72.6%)
Yes	141 (27.4%)
	**Mean ± SD**
Age (years)	27.55 ± 10.92
Household crowding index (persons/room)	1.15 ± .57
Body Mass Index (kg/m^2^)	24.27 ± 4.54
Avoidant restrictive eating	15.64 ± 8.48
Dysmorphic concerns	6.40 ± 5.12

### Bivariate analysis of factors associated with suicidal ideation

The results of the bivariate analysis of factors associated with suicidal ideation are summarized in Table 2. The results showed that a higher percentage of single vs married people had suicidal ideation. A lower mean age and higher mean avoidant restrictive eating and dysmorphic concerns scores were found in participants who had suicidal ideation vs not.

**Table 2:** Bivariate analysis of factors associated with wellbeing

Variable	Absence of suicidal ideation	Presence of suicidal ideation	*t / X*^*2*^	*df*	*p*
Sex			.10	1	.757
Male	114 (73.5%)	41 (26.5%)			
Female	260 (72.2%)	100 (27.8%)			
Marital status			4.76	1	**.029**
Single	264 (70.0%)	113 (30.0%)			
Married	110 (79.7%)	28 (20.3%)			
Education			2.57	1	.109
Secondary or less	67 (79.8%)	17 (20.2%)			
University	307 (71.2%)	124 (28.8%)			
Living area			.84	1	.358
Urban	221 (74.2%)	77 (25.8%)			
Rural	153 (70.5%)	64 (29.5%)			
Age	28.37 ± 11.68	25.36 ± 8.20	3.28	513	**.001**
Household crowding index	1.13 ± .54	1.23 ± .64	-1.80	513	.073
Body Mass Index	24.21 ± 4.51	24.43 ± 4.63	-.49	513	.626
Avoidant restrictive eating	15.01 ± 8.49	17.30 ± 8.28	-2.78	513	**.006**
Dysmorphic concerns	5.45 ± 4.68	8.90 ± 5.40	-6.69	513	** < .001**

Numbers in bold indicate significant *p* values

### Mediation analysis

**Fig. 1:**
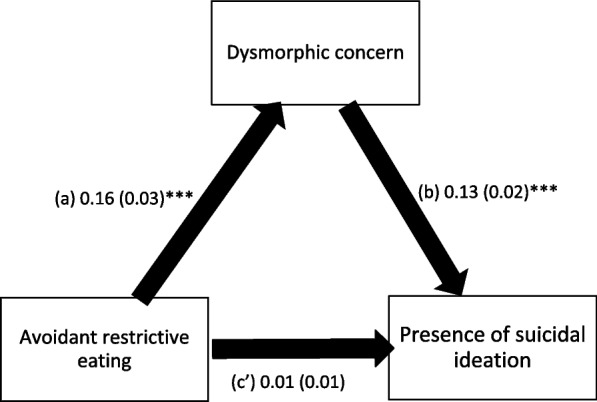
**a** Relation between avoidant restrictive eating and dysmorphic concern (R2 = .075); **b** Relation between dysmorphic concern and the presence of suicidal ideation (R2 = .154); **c**’ Direct effect of avoidant restrictive eating on the presence of suicidal ideation (R2 = .154). Numbers are displayed as regression coefficients (standard error). ***p < 0.001

The results of the mediation analysis are summarized in Table 3 and Fig. 1; the analysis was adjusted over the following variables: age, education, marital status, and household crowding index. Dysmorphic concerns fully mediated the association between avoidant restrictive eating and suicidal ideation. Higher avoidant restrictive eating was significantly associated with more dysmorphic concerns, and higher dysmorphic concerns were significantly associated with the presence of suicidal ideation. Finally, avoidant restrictive eating was not significantly associated with suicidal ideation.

**Table 3:** Mediation analysis results, taking avoidant restrictive eating as the independent variable, dysmorphic concern as the mediator and the presence/absence of suicidal ideation as the dependent variable

	**Direct effect**			**Indirect effect**		
	**Beta**	**SE**	***p***	**Beta**	**Boot SE**	**Boot CI**
Nightmares distress	.01	.01	.278	.02	.01	.01; .03^a^

^a^indicates significant mediation. Direct effect refers to the direct association between avoidant restrictive eating and the presence of suicidal ideation without the effect of the mediator, whereas the indirect effect refers to the same association through the mediator (dysmorphic concern)

## Discussion

Our study reveals a notable link between higher avoidant restrictive eating and dysmorphic concerns in agreement with the existing medical literature that has indicated a discernible association between ARFID and BDC [12]. This linkage stresses the potential interplay and shared psychological aspects between these two distinct yet interrelated conditions. While ARFID diagnostic criteria typically exclude body image distortions [5], individuals with ARFID symptoms may still exhibit body image concerns [12, 27]. An illustrative case study featured a nine-year-old with severe malnutrition, wherein the patient's positive perception of her small size and emphasis on thinness was influenced by social media beauty standards and societal praise. Notably, her avoidant and restrictive eating behaviors stemmed from low appetitive drive, apprehension towards new foods, and fear of adverse consequences from eating. Her body image concerns were not the primary driver of disordered eating [27]. In another case report, the patient, an 18-year-old female, presented with severe underweight and cachexia. Notably, she did not intentionally restrict her food intake, acknowledged her thinness, and did not express a fear of gaining weight. During her inpatient treatment for refeeding, the patient disclosed a long-standing "hatred of face," indicating an intense dissatisfaction with her facial appearance. Subsequently, she received diagnoses of both ARFID and BDC. This case exemplified a clinical instance where ARFID and BDC co-occurred, suggesting a potential interplay between difficulties related to food intake and body image perceptions [12]. Recent research suggests that there is a common co-occurrence of BDC alongside eating disorders [28]. This association could be explained by the fact that eating disorders (specifically anorexia nervosa) and BDC share similar patterns of abnormalities in visual processing and perceptual organization, including over-attention to details and reduced processing of larger global features. Indeed, brain imaging studies show abnormal brain activation in frontal, parietal, striatal, and visual systems for both disorders [29]. Moreover, our research highlighted a significant connection between higher dysmorphic concerns and suicidal ideation. A prior US-based study revealed a notable prevalence of lifetime suicidal ideation in individuals with BDC, standing at 78% [30]. A compilation of studies contributed to our understanding of the association between BDC and heightened suicidal ideation. One study elucidated a positive and statistically significant correlation between BDC and suicidality, revealing compelling odds for both suicide attempts and suicidal ideation among individuals grappling with BDC [16].

Moreover, in the context of clinical assessments conducted during admission to a hospital setting, it was discerned that major depressive episodes and BDC functioned as unique markers for heightened suicidality in acute psychiatric scenarios. This observation accentuated the unique role that BDC played in contributing to the overall risk profile of individuals with psychiatric concerns, warranting specialized attention and care [31]. Furthermore, an exploration into twin samples, identified through the Child and Adolescent Twin Study in Sweden, revealed an enduring link between BDC symptoms and various facets of suicidality. These associations maintained their strength even after accounting for depressive and anxiety symptoms, highlighting the independent impact of BDC on suicidal ideation and behaviors [15]. Limited research exists in the literature concerning the mechanisms that contribute to suicidal ideation in individuals with BDC. One study investigated the connection between BDC and the acquired capability for suicide. It indicated that variables such as Post-Traumatic Stress Disorder, major depressive disorder, work impairment over a lifetime due to BDC, and a history of BDC-related restrictive food intake were significant factors associated with the occurrence of suicidal ideation in individuals diagnosed with BDC [32].

Finally, no direct correlation was identified in our study, between avoidant restrictive eating and suicidal ideation proving that the link between ARFID and suicidal ideation is fully mediated by BDC, and that the relationship is only apparent in cases where dysmorphic concerns coexist. This finding contrasts with previous research, while they have demonstrated a robust correlation between eating disorders and suicidal ideation, none have directly associated ARFID with suicidal thoughts [9, 10]. Our study refuted a direct connection between ARFID and suicidal ideation. Moreover, considering earlier research highlighting the connection between ARFID and BDC, and the established link between BDC and psychological distress leading to an increased likelihood of harboring suicidal thoughts, it is evident that the observed higher levels of avoidant restrictive eating among participants showing suicidal ideation in our study can be attributed to the mediating effect of BDC.

### Clinical implications

The clinical implications of our study are significant in advancing the understanding of ARFID, its potential link to suicidal ideation, and the role of dysmorphic concerns as a potential mediator between them. Although a direct association between ARFID and suicidal ideation wasn't established in this study, the research illuminated the importance of dysmorphic concerns as a potential intermediary in this relationship. This finding suggests a potential avenue for intervention. One key takeaway for mental health professionals is the recognition of dysmorphic concerns as a potential target for intervention. By addressing these concerns, clinicians may have the opportunity to indirectly impact and potentially mitigate the risk of suicidal ideation among individuals with ARFID. Incorporating interventions that focus on improving body image perception and self-esteem could play a pivotal role in enhancing mental health outcomes for this specific population.

In practical terms, this newfound knowledge empowers mental health professionals to refine their assessment techniques, develop targeted interventions, and tailor treatment plans that address both ARFID and dysmorphic concerns. By recognizing the potential mediation of dysmorphic concerns and their impact on suicidal ideation, clinicians can offer more nuanced and effective care. Ultimately, these insights enhance the overall quality of care provided to individuals grappling with the complex interplay of ARFID, dysmorphic concerns, and suicidal ideation.

Finally, ARFID is a relatively new psychiatric (eating disorder) diagnosis, and research on this topic is newly emerging in all parts of the world. Yet, except for the validation study of the NIAS [17], there is no study on ARFID from the Arab world that can be identified. Being an unstudied phenomenon in the Arab countries and contexts, the exact way of how cultural factors interact with ARFID symptoms is still vague and unclear, especially since evidence suggests that ARFID can differ across food environments and cultures [33, 34]. Future national and cross-national studies are needed to explore the influence or Arab culture on manifestations of ARFID, and how BDC can interact with both ARFID and suicidal ideation across different cultural backgrounds.

### Limitations

While this study has provided valuable insights into the relationship between ARFID, dysmorphic concerns, and suicidal ideation, it is important to acknowledge a few potential limitations that could impact the interpretation of its findings. First, this is a cross-sectional study, and therefore, only captures a snapshot of data at a specific point in time. This limits the ability to establish causal relationships or determine the direction of the associations between ARFID, dysmorphic concerns, and suicidal ideation. This study might also not have addressed all possible confounding variables that could influence ARFID and suicidal ideation. In addition, the study’s sample was composed of a majority of females (69.9%), which could affect the findings, especially as evidence has shown that females are more likely to have suicidal ideation [2], and less likely to have ARFID [6] than males. Future studies need to use gender-proportionate samples to confirm the present findings. Finally, while the study suggests that dysmorphic concerns could mediate the association between ARFID and suicidal ideation, it does not investigate deeply the interconnected mechanisms of these variables. Further research is needed to uncover the underlying processes.

## Conclusion

In conclusion, this study investigated the potential link between ARFID, dysmorphic concerns, and suicidal ideation. While no direct association was identified between ARFID and suicidal ideation, the study revealed that dysmorphic concerns could play a crucial mediating role in this relationship. This suggests that individuals with ARFID might be at an elevated risk of experiencing suicidal ideation when they also exhibit dysmorphic concerns related to body image and perceived physical flaws. Thus, clinicians and mental health practitioners should be vigilant in assessing for dysmorphic concerns, especially when working with individuals diagnosed with ARFID, as this could serve as an indicator of heightened risk for suicidal ideation. Furthermore, these results underscore the complexity of mental health issues and the importance of considering multiple factors when evaluating and treating patients. Addressing dysmorphic concerns alongside ARFID could potentially lead to more effective interventions aimed at reducing suicidal ideation and promoting overall mental well-being.

Looking ahead, future perspectives could encompass the development of targeted interventions that focus on addressing dysmorphic concerns among individuals with ARFID. Incorporating cognitive-behavioral strategies, psychoeducation about body image, and self-acceptance techniques could potentially enhance therapeutic outcomes. Moreover, longitudinal studies tracking the progression of ARFID and its interaction with dysmorphic concerns over time could offer valuable insights into the temporal dynamics of this relationship.

## Data Availability

All data generated or analyzed during this study are not publicly available due the restrictions from the ethics committee, but are available upon a reasonable request from the corresponding author.
